# Autophagy Paradox: Strategizing Treatment Modality in Melanoma

**DOI:** 10.1007/s11864-023-01053-8

**Published:** 2023-01-21

**Authors:** Christian Pangilinan, Xiaowei Xu, Meenhard Herlyn, Chengyu Liang

**Affiliations:** 1 Molecular and Cellular Oncogenesis Program, The Wistar Institute, 3601 Spruce Street, Philadelphia, PA 19104 USA; 2 Department of Pathology and Laboratory Medicine, Perelman School of Medicine, University of Pennsylvania, Philadelphia, PA USA

**Keywords:** Skin cancer, Melanoma, Autophagy, Lysosome, Targeted therapy, Immunotherapy

## Abstract

The primordial autophagy process, originally identified as a starvation response in baker’s yeast, has since been shown to have a wide spectrum of functions other than survival. In many cases, it is accepted that autophagy operates as a key tumor suppressor mechanism that protects cells from adverse environmental cues by enforcing homeostasis and maintaining the functional and structural integrity of organelles. Paradoxically, heightened states of autophagy are also seen in some cancers, leading to the prevailing view that the pro-survival aspect of autophagy might be hijacked by some tumors to promote their fitness and pathogenesis. Notably, recent studies have revealed a broad range of cell-autonomous autophagy in reshaping tumor microenvironment and maintaining lineage integrity and immune homeostasis, calling for a renewed understanding of autophagy beyond its classical roles in cell survival. Here, we evaluate the increasing body of literature that argues the “double-edged” consequences of autophagy manipulation in cancer therapy, with a particular focus on highly plastic and mutagenic melanoma. We also discuss the caveats that must be considered when evaluating whether autophagy blockade is the effector mechanism of some anti-cancer therapy particularly associated with lysosomotropic agents. If autophagy proteins are to be properly exploited as targets for anticancer drugs, their diverse and complex roles should also be considered.

## Introduction

Melanoma is the malignancy of pigment-producing melanocytes of the skin and rarely, the uveal layer of the eye. Though constituting only a small proportion of all skin cancer types, melanoma is deemed as the most fatal form ranking among top 5 in the US 2022 estimated leading cancer cases and deaths [1]. Genetic alterations, primarily affecting the mitogen-activated protein kinase (MAPK) signaling pathway, have been identified in melanoma, majority of which is clustered on gain-of-function mutations in BRAF, a cell-signaling molecule that regulates the MAPK pathway [2]. Despite tremendous advancements with BRAF-targeted therapies and immune checkpoint inhibitors [3], the clinical benefit of these current treatment modalities for melanoma is curbed by the almost inevitable development of resistance and tumor relapse [4]. To date, although new molecularly targeted agents are being developed [5], most have only achieved partial clinical responses or work in a subset of patients, suggesting that melanoma pathogenesis is far more complex than the intrinsic genetic determinants. Indeed, a growing body of evidence indicates that non-genetic mechanisms also contribute to melanoma persistence, resistance, and/or recurrence, which operate in a broad range of biological processes, including metabolic reprogramming, phenotypic switching, and immune microenvironment remodeling [6]. Understanding the unique biology and evolution of melanoma over space and time is crucial to the development of more effective and durable therapies for melanoma treatment. It is within this context that we discuss some of the current contrasting paradigms on autophagy, a fundamental homeostatic process that often goes awry in melanoma, and its exploitations in melanoma therapy.

## Autophagy: a landscape beyond survival and death

**Figure 1:**
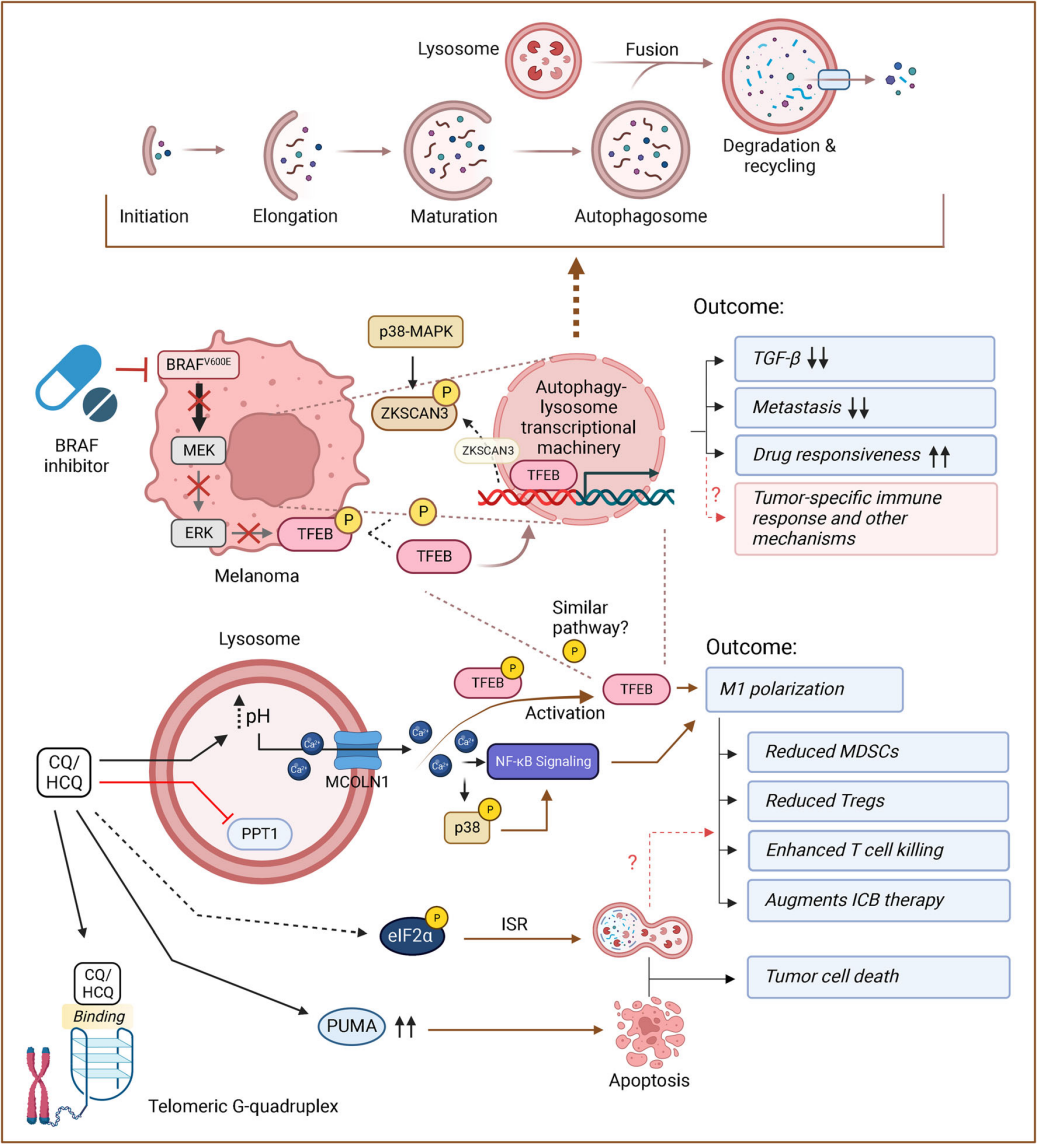
Dynamic roles of autophagy in melanoma. (1) Schematic representation of autophagy process. (2) BRAFi induces transcriptional activation of autophagy-lysosome program (by disrupting ERK-mediated TFEB phosphorylation and promoting p38-MAPK-mediated phosphorylation/inactivation of the autophagy repressor ZKSCAN3) and its blockade causes tumor progression, metastasis, and drug resistance that is associated with enhanced TGF-**β** signaling. (3) CQ modes of action in addition to inhibition of autophagy flux. This includes but not limited to TFEB activation and nuclear translocation (in part, by pH-dependent calcium release through MCOLN1), p38 and NF-κB signaling activation (following calcium release) and consequent macrophage polarization (from M2 to M1) and associated increased anti-tumor immunity, telomeric G-quadruplex binding, PUMA stabilization and induced apoptosis, as well as ISR activation (through eIF2**α** phosphorylation). MEK, mitogen-activated protein kinase; ERK, extracellular signal-regulated kinase; p38-MAPK, mitogen-activated protein kinase; TFEB, transcription factor EB; ZKSCAN3, zinc finger with KRAB and SCAN domains; CQ/HCQ, chloroquine/hydroxychloroquine; MCOLN1, Mucolipin-1; PPT1, Palmitoyl-protein thioesterase 1; NF-κB, nuclear factor kappa B; MDSCs, myeloid-derived suppressor cells; Tregs, regulatory T cells; ISR, integrated stress response; PUMA, p53 upregulated modulator of apoptosis. This figure was created with BioRender.com.

When the molecular machinery of autophagy was discovered 30 years ago using yeast genetics [7], autophagy was little more than just one interesting process of cells in response to nutrient deprivation. An explosive interest in autophagy biology is driven by the recognition that autophagy is associated with almost every facet of human disease including cancer [8]. Autophagy is generally classified as three mechanistically distinct forms that share the same terminal station of lysosomes: chaperone-mediated autophagy (CMA), microautophagy, and macroautophagy [9]. CMA engages the selective translocation and degradation of protein substrates containing a consensus KFERQ-like motif that can be recognized by chaperone complexes [10]. Microautophagy involves the engulfment of cytosolic protein cargoes through direct invagination of late endosomal and/or lysosomal membranes, which is recently found to be executed by the endosomal sorting complex required for transport (ESCRT) machinery [11]. Macroautophagy is hallmarked by the formation of the classic double-membrane-bound autophagosomes that sequester cellular cargo often but not always tagged with ubiquitin [12]. Autophagosomes are then progressively acidified by sequential fusion with endosomes and the lysosomal compartment to form autolysosomes, eventually leading to the degradation of encapsulated contents by lysosomal hydrolases (Fig. 1). Relative to other autophagy types, macroautophagy (hereafter referred as autophagy) is salient in maintaining homeostasis and damage control because the large capacity of autophagic capsules engenders the elimination of unwanted cellular components (e.g., microbes) and the turnover of key organelles such as mitochondria, endoplasmic reticulum, and ribosomes [8]. Over 40 autophagy-related (ATG) genes have been identified so far in yeast, and more numbers of ATG genes or genes with ATG functions are probably expressed in mammals and participate in the various stages of autophagy, which have been extensively reviewed elsewhere [13]. This complex protein network detects intracellular perturbation, signals this detection, and clears cytosolic abnormalities through the autophagosome-lysosome pathway for degradation and recycling. The most well-studied autophagy-inducing perturbation is nutrient deprivation, whereby increased adenosine monophosphate to adenosine triphosphate (AMP:ATP) ratio is sensed by AMP-activated protein kinase (AMPK) [14]. AMPK activation phosphorylates its downstream ATG effectors including unc-51 like autophagy activating kinase 1 (ULK1), Beclin 1, and ATG9 to mount autophagy, while concomitantly dampens mammalian target of rapamycin complex 1 (mTORC1)-mediated anabolic process [14]. mTORC1 suppression also promotes the activation and nuclear translocation of transcription factor EB (TFEB) to prime the expression of the autophagy-lysosome gene network, which further enhances induction of an autophagy response [15]. Notably, the spectrum of autophagic responses in mammalian cells is not limited to nutrient deprivation but widely adapts to a broad range of environmental and internal perturbations. When cells are challenged by infection, tissue damage, hypoxia, genotoxic agents, radiation, or other unfavorable conditions, autophagy restrains cell growth and enforces the defense of homeostasis through activities that eradicate harmful agents, promote genomic and organelle integrity, sustain oncogene-induced senescence, and/or arrest the cell cycle [16]. While excessive autophagy is often considered to be cytotoxic and can induce a form of cell death, termed autophagic cell death, the morphology of vast accumulation of autophagosomes before or during cell death is more generally considered to be a failed commitment of “self-defense,” rather than a direct executor of death program [17, 18]. On the other hand, basal autophagy, that operates constitutively at low levels in the absence of perturbations, is vital for cellular homeostasis and normal cell differentiation [19]. Although the exact mechanisms by which impaired functions of autophagy affect tissue homeostasis and their implications in disease are far from understood, autophagy, being an integral part of cell biology, should not be simply deduced into one-sided survival or death program.

Importantly, increasing evidence now reveals that, in addition to their role in regulating autophagy, many, if not all, of the molecular mediators of autophagy have functions outside of the autophagy pathway [20]. For instance, Beclin 1, a core subunit of the phosphatidylinositol 3-OH kinase class III complex (also known as PI3KC3) required for autophagosome nucleation, is broadly involved in vesicular trafficking [21]. UV radiation resistance-associated gene (UVRAG), a bona fide Beclin 1 activator in autophagy, also serves as a gatekeeper of chromosomal stability and promotes UV-induced photolesion repair independently of autophagy [22, 23]. Along similar lines, ATG7, an E1-like enzyme in autophagy-related conjugation reaction, can bind the all-important tumor suppressor p53 and regulate p53-mediated cell cycle arrest and apoptosis irrespective of degradative autophagy [24]. These non-autophagic behaviors of ATG gene products reflect the crosstalk between autophagic responses and multiple other cellular processes [20], but could also confound the interpretation of their functions more biased toward autophagy-relevant domains when overlooked. For instance, conditional deletion of autophagy/beclin-1 regulator 1 (*Ambra1*), a positive regulator of Beclin 1-dependent autophagy [25], resulted in accelerated cell proliferation and subsequent cell death in the developing neuroepithelium, which is recently found to be caused by increased levels of cyclin D [26]. In fact, AMBRA1 mediates the proteasomal turnover of cyclin D as a substrate receptor for the cullin 4-based E3 ligase complex, a mechanism that contributes to cancer development and chemoresistance [27**•**]. A comprehensive understanding of these novel properties of autophagy regulators could aid in the development of new strategies to precisely prevent and treat multiple autophagy-related pathologies.

## Autophagy and melanoma: a longstanding paradox between good and evil

Owing to its key roles in the preservation of cellular homeostasis, autophagy in most cases constitutes a critical barrier against malignant transformation of healthy cells [16]. In this light, it was not surprising to find that proteins with oncogenic potential generally suppress autophagy, whereas many oncosuppressive ones promote autophagic response [16, 28]. Recent findings of autophagy in several aspects of anti-tumor immunity further support this view [28, 29]. However, despite the inhibitory role in tumor suppression, it has been increasingly realized that autophagy also fosters tumor growth, metastasis, and even therapy resistance, principally through its actions to sustain the survival of metabolically stressed neoplastic cells [30]. Conflicting results regarding the contribution of autophagy to the biology of cancer reflect our incomplete understanding of molecular regulation and execution of autophagy in different contexts [28]. It is not understood what makes the “pathological autophagy” process different from “homeostatic autophagy,” though both seem to engage the similar core autophagy machinery. Does the pathological mode of action represent a functional continuity of homeostatic autophagic response but with altered magnitude, or maybe it involves distinct gene expression program and downstream effector mechanism? Understanding the mechanisms underlying tumor-elicited or tumor-rewired autophagy (i.e., the autophagy reaction following tumor development) may help settle the “autophagy paradox” prevalent in malignant disease including melanoma as outlined below.

While in general, ATG gene mutations are rare in cancer, data inferred from a large cohort of human melanoma patients revealed low expression levels of ATG5, a critical factor for autophagosome membrane expansion, in primary tumors compared to benign nevi, which correlates with impaired autophagic activity and reduced progression-free survival in patients [31]. Moreover, ectopic expression of ATG5 inhibits the colony-forming ability of melanoma cells, whereas its downregulation can bypass oncogene-induced senescence, at least in vitro, implying that less autophagy means more transformation [31]. In line with this notion, additional findings showed that hemizygous loss of *ATG5* occurred during melanoma progression, and reduced expression of ATG5 and ATG7 was observed in both primary and metastatic melanoma tissues likely due to deficiency of nuclear respiratory factor (NRF1) [32, 33]. As further corroboration of these results, deletion of *Atg7* significantly accelerated melanoma onset and worsened overall survival in melanoma murine model with melanocyte-specific expression of oncogenic BRAF^V600E^, which was overridden upon *Pten* deficiency, highlighting a critical barrier function of autophagy for melanoma development [34]. At apparent odds with these findings, work by Xie and colleagues showed that *Atg7* ablation failed to promote, but rather impeded the development of melanoma driven by BRAF^V600E^ and allelic *Pten* loss and extended mouse survival [35]. The simplest interpretation of this observation was that the loss of Atg7 and by extension autophagy could prevent melanoma, implying that basal autophagy could contribute to tumorigenic process in certain context. Although autophagy loss in this case was also associated with increased DNA damage, dysfunctional mitochondria, and accumulated p62, consistent with its traditional role in preserving organelle integrity [35], the exact mechanisms that link ATG7 to oncogene-driven melanoma are not well-defined. It is unclear whether these observations indicate essential roles of basal autophagy per se in sustaining neoplastic transformation or, rather, alternative functions of ATG proteins in cell death signaling or execution at play. Particularly, recent studies suggest that the outcome of suppressing autophagy in tumor development depends on the status of p53; in the absence of intact p53, loss of autophagy or the use of autophagy inhibitor accelerates tumorigenesis [36]. Hence, one likely explanation is that the sublethal cellular stress induced by absence of ATG7 in oncogene-primed melanocytes might be recognized by p53, which then attempts to kill the cell by inducing apoptosis, preventing further proliferation and/or transformation; and if so, tumor suppression occurs through a p53 pathway but not necessarily by autophagy deficiency. Nevertheless, most reports so far acknowledge autophagy as a vital suppressive process against melanomagenesis, and the suppressive nature is largely attributed to autophagy-mediated cell-intrinsic protection against oxidative stress, DNA damage, and/or organelle stability [16].

Despite the ambiguous roles of autophagy in melanoma development, data is emerging showing that autophagy was consistently induced in melanoma patients who were given highly specific BRAF^V600E^ inhibitors (BRAFi) [37, 38]. So what does this induced autophagy have to do with melanoma and therapy? Ma and co-workers proposed that BRAFi treatment promoted autophagy through a protein kinase R-like endoplasmic reticulum kinase (PERK)-dependent ER stress response, and that elevated autophagy correlated with poor therapeutic response [37]. This finding logically led to the conclusion that increased autophagy in melanoma goes hand in hand with therapy resistance, and thus in short, should be targeted [37]. However, the story soon became more complicated when it was found that it was oncogenic BRAF (but not BRAF inhibition) that induces chronic ER stress that could sensitize melanoma to apoptosis [39]. Along this line, a recent study revealed that elimination of PERK-mediated ER stress potentiated paraptosis-associated immunological cell death in stressed melanoma cells [40]. Although theoretically autophagy can provide adaptive survival advantage of ER-stressed cells, its inhibition does not seem to confer an anti-tumor effect in PERK-null melanomas [40]. Any interpretation of the potential role of autophagy in melanoma therapy requires a pre-understanding of how autophagy is regulated and abnormally functions in cells. In this light, more recent work in our and other laboratories has led to the discovery that, upon exposure to BRAFi, melanoma cells mobilize autophagy, surprisingly not through the previously mentioned induction of ER stress, but through activation of TFEB, a master regulator of the autophagy-lysosome network (Fig. 1) [41••, 42]. Removing TFEB erased the autophagy-promoting effect of BRAFi irrespective of ER stress machinery [41••]. Further analyses revealed mTORC1-independent TFEB phosphorylation and inactivation by constitutively activated ERK in BRAF^V600E^ melanomas, which can be reversed by BRAFi [41••]. Although it is generally thought that a tumor with higher autophagic response would respond worse to chemotherapy, using a xenograft model, it was found that TFEB-activated tumors actually had higher sensitivity to BRAFi as measured by both clonogenic survival and regrowth of the tumors following treatment [41••]. Consistently, TFEB-disrupted clones, which showed decreased autophagy-lysosome activity, displayed higher resistance to BRAFi treatment and increased treatment-associated metastasis and dedifferentiation [41••], suggesting that impaired autophagy-lysosomal response may be causally related to drug resistance in at least in BRAF^V600E^ melanomas. How impaired autophagy translates into more BRAFi resistance was found in the upregulation of transforming growth factor β (TGF-β), a direct mediator of cell dedifferentiation and metastasis, that was found to be aberrantly accrued in cells lacking functional TFEB [41••]. Although it remains to be shown whether BRAF^V600E^ targets TFEB in other cellular processes, these observations that tumors with the least autophagy-lysosome activities could become most aggressive suggest that, at least in certain context, the oncosuppressive functions of autophagy may prevail over the prosurvival effects of autophagy in limiting melanoma progression, as seen in other cancers [43]. Similar observations were recently made by Marsh and co-workers showing that autophagy inhibition enriched highly aggressive cells that exhibited an increased propensity for metastasis [44•]. With those new insights, we might also have to rethink the applicability of autophagy inhibitors in patients receiving targeted therapy for melanoma and other relevant cancers. Although the prosurvival aspect of autophagy makes it an attractive target for cancer therapy, blanket inhibition of autophagy could also subvert treatment response by means of for instance TGF-β activation and/or tumor dedifferentiation, which might be particularly a concern for tumors with high plasticity and phenotypic diversity [6]. Although certain more data is needed to support these findings, their mere possibility is challenging for researchers and pharmaceutical companies alike.

## Melanoma autophagy and immunosurveillance: a new battlefield for fire up or for escape

Although much of the original experimental studies have been centered on cell-intrinsic mechanisms of autophagy in cancer suppression or progression, the stage has now clearly been set for autophagy operation in remodeling tumor microenvironment and/or reshaping immune landscape that modulate malignancy [45]. Theoretically, this may ultimately be clinically useful in conjunction with immune checkpoint blockade (ICB). Being a critical prerequisite to preserve homeostasis and protect against perturbations for almost every cell type, it is not surprising to find that autophagy is required for the normal differentiation and function of both myeloid and lymphoid cells [45]. Germline mutations in ATG genes are associated with immune-relevant pathologies [29, 45]. Systemic stimulation of autophagy by time-restricted fasting or fasting mimetics resulted in improved immunosurveillance and thereof reduced tumor burden either standalone or in combination with immunogenic chemotherapy or ICB [46–48]. Thus, in general, autophagy is considered as an immune booster, not only because it helps to present the proper antigenic profile of antigen-donor cells but also is required for the release of immunogenic signals (e.g., ATP) to elicit cognate immune response [29, 45]. However, as seen with its intrinsic impact on tumor growth, roles of autophagy in the tumor-immune interface also exhibits a significant degree of context dependency and sometimes is even turned upside down. Interestingly, using in vitro functional assays and syngeneic animal models, Yamamoto and co-workers studied pancreatic ductal adenocarcinoma (PDAC) expressing a dominant-negative mutant of ATG4B and found that, PDAC tumors with autophagy ablation exhibited increased cytotoxic T cell infiltration in the tumor bed and subsequently restricted tumor growth [49**••**]. Further study revealed that PDAC tumors were able to downregulate surface major histocompatibility complex (MHC-I) molecules through an autophagy-dependent process [49**••**]. Although the generality of this finding remains to be tested, it provides plausible mechanistic explanations for the ability of some tumors to promote immune evasion via autophagy at least in certain circumstances. Moreover, autophagy was found to act as a negative regulator of stimulator of interferon genes (STING)-dependent type I interferon secretion through improved mitochondrial DNA clearance in irradiated cancer cells [50], irrespective of its established role in attenuating tumor-promoting inflammation [29]. Nonetheless, the confusing results of tumor-specific autophagy in different experimental animal models make us perceive that, while successful autophagic response ensures a well-balance immunosurveillance, it might be skewed in different way by different tumors for their immune evasion that should be carefully considered in cancer immunotherapy.

That said, melanoma is considered to be an immunogenic cancer that shows high responsiveness to ICB. Still so, up to two-thirds of patients do not respond. Although the causes of immune resistance remain elusive, insufficient release or presentation of tumor antigens is considered to be one major factor, a domain of autophagy at play [51]. Notably, retrospective cohorts analysis of melanoma patients treated with cytotoxic T-lymphocyte associated protein 4 (CTLA-4) inhibitors revealed a unique signature of cancer-germline antigens that could predict resistance uniquely to blockade of CTLA-4 (not programmed cell death protein 1 or PD-1), and that was associated with autophagy suppression in tumors [52]. Although further analyses are certainly needed to understand the mechanisms of autophagy attenuation in these resistant tumors, this study provided the clinical evidence to the importance of the autophagy pathway in modulating anti-tumor T cell immunity. Remarkably, similar conclusion was reached using preclinical models, showing that loss of PTEN, which activates the PI3K-AKT (also known as protein kinase B) pathway, promotes resistance to T-cell-mediated immunotherapy by autophagy suppression [53]. Furthermore, enforced expression of ATG genes restored susceptibility of melanoma cells to T cell-mediated cell killing, whereas its inhibition by hydroxychloroquine (HCQ) caused resistance [53]. These studies, along with other findings of melanoma-specific autophagy in promoting immunogenic cell death [54–56], suggest that autophagy deficiency may serve as a mechanism that drives the establishment of “innate” immune escape of at least some fractions of melanomas. Going back to our previous report [41**••**], TFEB reactivation-associated autophagy-lysosomal activation attenuated TGF-β signaling — a known immunosuppressive mechanism — which may synergize with various immunotherapeutic and potentiate antitumoral T cell response. Notwithstanding the prior limited efficacies of anti-TGF-β agents in clinical trials as monotherapy [57], encouraging data are now emerging demonstrating that the combination of TGF-β inhibitors with ICB can eradicate drug-resistant tumors [58, 59]. As expected, the matters can only become more complicated when it comes with different models used in different laboratories. Mgrditchian et al. [60] used syngeneic melanoma B16-F10 model and found that inhibition of autophagy (by *Beclin 1* depletion) resulted in a C-C motif chemokine ligand 5 (CCL5)-mediated NK cell homing at the tumor sites, which might contribute to reduced tumor volume. However, the significance and mechanism of action of this finding need to be uncovered, particularly in consideration of the essential play of autophagy in NK cell-induced innate immunity [61].

After all is said and done, however, and despite these advances in knowledge of tumor-intrinsic autophagy in anti-tumor immunity, the dynamic roles of this multifaceted autophagic process in the “tug-of-war” with host immunity, for the moment at least, unclear.

## Autophagy and melanoma therapy: a caution and a misnomer

The centrality of autophagy in both cancer biology and immunology makes it an intriguing target for cancer therapy development. No wonder many efforts have been undertaken over the past decades, both in academic research and industrial drug discovery, to develop specific autophagy-based anticancer treatments that can be used as adjuvants or stand-alone agents. This turns not to be a simple task, in part because autophagy, as both a response and a process, is intimately connected to other biological processes, as well as the fact that many autophagy-relevant proteins carry non-autophagic roles that could easily confound many therapy-related data interpretation [20]. Whether autophagy should be inhibited or activated is highly debated in the cancer community and its targeting is likely cancer-dependent.

Thus far, the most widely reported approach for autophagy inhibition which has potential clinical applicability has been the use of lysosomotropic agents, such as chloroquine (CQ) — the known antimalarials [62]. Because such agents neutralize the lysosomal pH and thereof inhibit the action of lysosomal hydrolases, it can attenuate most, if not all, membrane trafficking routes that terminate or transit at lysosomes, including but certainly not limiting to autophagy. While CQ-like agents can serve as “autophagy inhibitors,” they possess a variety of properties beyond autophagy that make this appellation, in many cases, a confusing misnomer (Fig. 1). For instance, contrary to the conventional view, the cytotoxic effects of CQ were found not to be autophagy-dependent but related to lysosomal membrane permeabilization, which can trigger autophagy-independent cell death [63, 64]. Meanwhile, increased pH in acidic compartments increases the intracellular retention of some chemotherapy drugs, thereby sensitizing tumors to these treatments [65]. Moreover, CQ and/or its derivatives are also found to target telomeric G-quadruplex [66] and palmitoyl-protein thioesterase 1 (PPT1) [67], induce nuclear factor kappa B (NF-κB) signaling activation and macrophage M2-to-M1 polarization [68, 69], and enhance antigen presentation by dendritic cells through endolysosomal de-acidification [70]. The discovery that CQ could directly induce apoptosis through a pro-apoptotic PUMA-dependent, but not a lysosomal protease-dependent, fashion exclusive to melanoma is intriguing [71], adding another level of complexity to CQ actions. With those new insights, CQ-associated anti-tumor activities and CQ-based trials should be rigorously evaluated. This notion is further strengthened by the work of Eng and co-workers [72], showing that deletion of ATG genes failed to recapitulate the antiproliferative effects of CQ in tumors such as those with oncogenic RAS mutations. More interesting twists to the CQ-autophagy story came when it was found that CQ treatment actually triggers eIF2α phosphorylation, a bona fide ignitor of the autophagy process [73], as well as previously realized activation of TFEB and TFE3, which transcriptionally upregulate most of the autophagy-lysosomal genes and consequently inhibit mTORC1 activity [74]. Thus, it seems that by transiently perturbing lysosome integrity and function, the ultimate consequence of CQ seem to induce, rather than suppress, an integrated stress response including autophagy [73]. Clearly, more specific autophagy modulators are truly needed to sharpen our experimental setting and broaden clinical exploration. Alternative strategy is being centered on the development of small molecule compounds that more specifically target the autophagic machinery in cells such as those inhibiting the initiating kinase ULK1, PI3KC3 (Vps34), the proteolytic enzyme ATG4, and the enzymes required in autophagy conjugation system (Table 1) [75]. Despite numerous reports claiming the benefit of autophagy inhibition in cancer therapy, great caution should be taken before translating in vitro even preclinical data into clinical use, particularly considering its deleterious effects for post-mitotic cells [76, 77].

**Table 1:** Autophagy-modulating agents under investigation for melanoma other than lysosomotropic agents

**Agent**	**Action**	**Autophagy-related mechanism**	**Refs**
SAR405	Class III PI3K (Vps34) inhibitor	Inhibit autophagosome formation	[81]
3-MA	Class III PI3K (Vps34) inhibitor	Inhibit autophagosome formation	[82]
DCC-3116	ULK1/2 inhibitor	Inhibit initiation of autophagy process	Deciphera Pharmaceuticals
Trifluoperazine	Anti-schizophrenic drug	Inhibit autophagy flux	[83]
Salinomycin	N/A	Inhibit autophagic flux	[84]
Mdivi-1	Dynamin-related protein (Drp1) GTPase inhibitor	Inhibit mitophagy	[85]
Caloric restriction mimetics (CRM)	Multiple	Activate autophagy initiation signals	[46]
GSK621	AMPK activator	Activate autophagy initiation signals	[86]
Everolimus (RAD-001)	mTORC1 inhibitor	Activate autophagy initiation signals	[87]
Resveratrol	CRM	Activate autophagy initiation signals	[88]
IMD-0354	Inhibit glutamine uptake and mTORC1 signaling	Activate autophagy initiation signals	[89]
C2-ceramide	Phosphatase-associated proteins (CAPP) activator	Activate autophagy initiation signals	[90]
Trehalose	Akt inhibitor	Activate TFEB-induced autophagy-lysosome program	[91]
Lithium	Reduce Ins(1,4,5)P3 and inositol levels	Activate phagophore nucleation	[92]
Metformin	Reduce tribbles Pseudokinase 3 (TRIB3) expression	Activate autophagic flux	[93]
Capsaicin	NADH oxidase	Induce reactive oxygen species (ROS)-dependent autophagy	[94]
Melatonin	ROS reduction	Activate ER-stress induced autophagy	[95]
Dendrogenin	Liver X receptor agonist	Transcriptional induction of autophagy	[96]
Cannabinoid	N/A	Enhance autophagy-associated cytotoxicity	[97]
β-β-Dimethylacrylshikonin	N/A	Activate autophagy-associated cytotoxicity	[98]
Hernandezine	N/A	Activates autophagy-associated cytotoxicity	[99]

For similar aforenoted reasons, the search for pharmaceutical agents to specifically activate autophagic process has not, as yet, yielded many players. The use of small peptides or small molecule inhibitors to modulate the interaction of key autophagy proteins with their inhibitory partner has enormous potential, albeit not yet been applied in cancer treatment including melanoma. The best documented successful attempt in this regard was made by the team in Dr. Levine laboratory; this led to the development of a peptide derived from a region of Beclin 1 that interacts with HIV-1 protein Nef [78]. This Beclin 1-activating peptide displayed beneficial effects in alleviating pathologies associated with myocardial ischemia reperfusion, cardiac senescence, and neurodegeneration through autophagy stimulation [76–78]. A similar approach led to the identification of a short peptide derived from FLICE-like inhibitor protein (FLIP), an inhibitor of ATG3, which can unleash ATG3 from FLIP inhibition and induce massive cell death of virus-associated primary effusion lymphoma through autophagy activation [79]. Although it is too early to know whether these agents or their improved analogs will enter into the clinic eventually, the autophagy-inducing Beclin 1 peptide showed promising effects in suppressing breast cancer growth comparable to a clinically used HER2 tyrosine kinase inhibitor in xenograft models [80]. On a side note, these types of agents also constitute promising research tools, serving as a “clean” means to activate autophagy without imposing broad-ranging cellular chaos. In the meantime, novel therapeutic agents, and some established drugs (e.g., metformin) exert their robust anti-melanoma effects, though, at least in part, autophagy activation (Table 1).

Notwithstanding the caveats and/or specificity concern, it is necessary to keep in mind that future cancer therapies employing autophagy modulation will likely focus on the oncosuppressive (rather than pro-tumor), on the immunostimulatory (rather than immune evasive) spectrum of the autophagic response, and that effective autophagy-targeting therapies will result in transient and partial attenuation (rather than sustained and whole-body eradication) in cancers whereby the mechanistic basis is properly established in different tumor states.

## Conclusion and perspectives

Due to the almost inevitable occurrence of tumor resistance, relapse, persistence, and/or metastasis in melanoma, the search for new molecular targets is in high gear. Being an integral defense system essential for maintaining homeostasis, autophagy has come to the forefront as a major player in almost all kinds of human diseases including melanoma. Because stress-induced pro-survival autophagy is the most primordial and most studied forms of the response, we may have skewed our understanding of autophagy toward some extreme conditions which in many cases are not representative. With many important functions of autophagy already linked to different aspects of cancer prevention/genesis, regression/progression, resistance/response, and persistence/clearance, and with more likely to be discovered, one of the challenges that remains is the integration of these various components into a cohesive *big-picture* for what happens when autophagy encounters cancer and vice versa. Furthermore, systematic exploration of the debated aspects of autophagy in cancer and elucidation of the molecular mechanisms in action of autophagy-modulating agents should yield deeper understanding of autophagy-associated pathophysiology of diseases and innovate better therapeutic approaches. The future will tell whether autophagy-based therapy will indeed make a considerable impact on some aggressive malignancies like melanoma.
